# Supplementary material for: Broad Environmental Tolerance for a Salicola Host-Phage Pair Isolated from the Cargill Solar Saltworks, Newark, CA, USA
Source: Microorganisms. 2019 Apr 19;7(4):106. doi: 10.3390/microorganisms7040106 (PMC6518143; doi:10.3390/microorganisms7040106)
Supplement: Supplementary file 1 [file microorganisms-07-00106-s001.pdf]

Table S1. Substrate usage of the CGM5-S host determined using BIOLOG Phenotype Microarray plates.

Carbon Source	Carbon Source
L-Proline	Adenosine
D-Alanine	Citric Acid
Glycerol	M-Inositol
D-Glucoronic Acid	Fumaric Acid
D, L-α-Glycerol-Phosphate	Bromo Succinic Acid
L-Lactic Acid	Propionic Acid
Formic Acid	Glyoxylic Acid
D-Mannitol	Glyoxylic Acid
L-Glutamic Acid	D-Cellobiose
Tween 20	Glycyl-L-Glutamic Acid
Acetic Acid	L-Serine
α-D-Glucose	L-Threonine
L-Asparagine	L-Alanine
D-Aspartic Acid	L-Alanyl-Glycine
D-Glucosaminic Acid	Acetoacetic Acid
Tween 40	N-Acetyl-β-D-Mannosamine
α-Keto-Butyric Acid	Mono Methyl Succinate
α-D-Lactose	Methyl Pyruvate
Sucrose	D-Malic Acid
L-Glutamine	L-Malic Acid
M-Tartaric Acid	Glycyl-L-Proline
Glucose-1-Phosphate	P-Hydroxy Phenyl Acetic Acid
Fructose-6-Phosphate	Tyramine
Tween 80	D-Psicose
α-Hydroxy Glutaric Acid-γ-Lactone	Glucoronamide
α-Hydroxy Butyric Acid	Pyruvic Acid
β-Methyl-D-Glucoside	L-Galactonic Acid-γ-Lactone
Adonitol	D-Galacturonic Acid
Maltotriose	Phenylethylamine
2-Deoxy Adenosine	2-Aminoethanol
Butyric Acid	L-Alaninamide
Capric Acid	L-Histidine
β-Hydroxy Butyric Acid	

Table S2. Comparison of selected substrate usage for CGM5-S and described *Salicola*.[1.]

Substrate	CGM5-S (USA)	9-A-U (Mexico)	*S. marasensis* (Peru)	*S. salis* (Algeria)	7SPE isolates (Tunisia)	TBZ isolates (Iran)
Sugars						
Sucrose	+	-	+	-	NR	NR
α-D-Glucose	+	+	+	-	0/6	0/3
α-D-Lactose	+	+	+	-	NR	NR
D-Mannose	-	+	+	-	0/6	0/3
Alcohols						
D-Mannitol	+	+	+	-	NR	NR
Glycerol	+	+	+	NR	0/6	NR
M-Inositol	+	-	NR	NR	0/6	NR
Adonitol	+	-	NR	-	NR	NR
Amino Acids						
L-Arginine	-	+	+	-	6/6	NR
L-Tyrosine	NR	NR	NR	-	NR	0/3
L-Lysine	-	+	+	-	6/6	NR
Ornithine	NR	NR	+	-	6/6	NR
L-Alanine	+	+	+	-	NR	NR

Glycine	-	-	+	-	NR	NR
Polysorbates						
Tween 20	+	+	NR	NR	NR	3/3
Tween 40	+	+	NR	NR	NR	NR
Tween 80	+	+	NR	+	NR	3/3
Acids						
Malic Acid	+ (D-form)	+ (L-form)	+	NR	2/6	NR
Capric Acid	+	+	NR	NR	0/6	NR
Lactic Acid	+	+	+	-	NR	NR

[1] CGM5-S (this study); 9-A-U [30]; S. marasensis [25]; S. salis [72]; 7SPE isolates [71]; TBZ isolates [73]. NR = not reported. For 7SPE and TBZ isolates #/# = number isolates utilizing substrate/total number of isolates.

Table 3. Coding sequence annotations of CGϕ29 phage.

Start (nt)	Stop (nt)	Locus Tag	Direction	Gene Product Name
1	934	SLPG_00001	–	helicase
934	1881	SLPG_00002	–	hypothetical protein
1968	2954	SLPG_00003	–	DNA methylase
2998	3495	SLPG_00004	–	protein of unknown function (DUF669)
3498	3893	SLPG_00005	–	hypothetical protein
3894	4156	SLPG_00006	–	hypothetical protein
4153	4827	SLPG_00007	–	AAA domain
4954	5139	SLPG_00008	+	DNA-binding transcriptional regulator Cro
5132	7126	SLPG_00009	+	uncharacterized protein conserved in bacteria
7369	7668	SLPG_00010	+	hypothetical protein
7764	7937	SLPG_00011	+	protein of unknown function (DUF1391)
7892	8239	SLPG_00012	+	hypothetical protein
9047	9448	SLPG_00013	+	helix-turn-helix domain of resolvase
9423	10772	SLPG_00014	+	terminase large subunit
10773	12139	SLPG_00015	+	domain of unknown function (DUF4055)
12180	12926	SLPG_00016	+	hypothetical protein
13033	14187	SLPG_00017	+	P22 coat protein - gene protein 5
14395	15324	SLPG_00018	+	transposase IS116/IS110/IS902 family
15290	15490	SLPG_00019	+	hypothetical protein
15822	16001	SLPG_00020	+	hypothetical protein
15977	16483	SLPG_00021	+	hypothetical protein
16483	16845	SLPG_00022	+	hypothetical protein
16849	17880	SLPG_00023	+	phage Mu protein F like protein
17822	18292	SLPG_00024	+	hypothetical protein
18289	18516	SLPG_00025	+	hypothetical protein
18513	18737	SLPG_00026	+	hypothetical protein
18734	19129	SLPG_00027	+	bacteriophage related domain of unknown function
19183	19482	SLPG_00028	+	hypothetical protein
19479	19772	SLPG_00029	+	hypothetical protein
19754	19963	SLPG_00030	+	hypothetical protein
20147	20602	SLPG_00031	+	hypothetical protein
20616	20963	SLPG_00032	+	hypothetical protein
21057	21473	SLPG_00033	+	hypothetical protein
21500	21928	SLPG_00034	+	hypothetical protein
21984	26240	SLPG_00035	+	hypothetical protein
26237	27136	SLPG_00036	+	carbohydrate binding domain
27137	28825	SLPG_00037	+	FOG: WD40 repeat
28829	29641	SLPG_00038	+	phage Tail Collar Domain
29644	30711	SLPG_00039	+	hypothetical protein
30723	31124	SLPG_00040	+	hypothetical protein
31136	31387	SLPG_00041	+	hypothetical protein
31544	32107	SLPG_00042	+	peptidase family M23
32326	32514	SLPG_00043	+	hypothetical protein

32504	32719	SLPG_00044	+	hypothetical protein
32649	32819	SLPG_00045	+	hypothetical protein
32926	33105	SLPG_00046	–	hypothetical protein
33199	33462	SLPG_00047	–	hypothetical protein
33459	33674	SLPG_00048	–	hypothetical protein
33674	34516	SLPG_00049	–	hypothetical protein
34547	34978	SLPG_00050	–	hypothetical protein
34959	35282	SLPG_00051	–	hypothetical protein
35279	35440	SLPG_00052	–	hypothetical protein
35488	34694	SLPG_00053	–	hypothetical protein
35804	36007	SLPG_00054	–	hypothetical protein
36011	36223	SLPG_00055	–	hypothetical protein
36220	36678	SLPG_00056	–	hypothetical protein
36675	36932	SLPG_00057	–	hypothetical protein
36929	37480	SLPG_00058	–	hypothetical protein
37490	37672	SLPG_00059	–	hypothetical protein
37665	38360	SLPG_00060	–	protein of unknown function (DUF3310)
38350	38640	SLPG_00061	–	hypothetical protein
38640	38978	SLPG_00062	–	hypothetical protein
39049	39471	SLPG_00063	–	VRR-NUC domain
39513	40691	SLPG_00064	–	helicase conserved C-terminal domain
